# Genomic variants identified from whole-genome resequencing of indicine cattle breeds from Pakistan

**DOI:** 10.1371/journal.pone.0215065

**Published:** 2019-04-11

**Authors:** Naveed Iqbal, Xin Liu, Ting Yang, Ziheng Huang, Quratulain Hanif, Muhammad Asif, Qaiser Mahmood Khan, Shahid Mansoor

**Affiliations:** 1 National Institute for Biotechnology and Genetic Engineering (NIBGE), Faisalabad, Punjab, Pakistan; 2 Beijing Genomic Institute (BGI), Shenzhen, Guangdong, China; 3 Department of Biotechnology, Pakistan Institute of Engineering & Applied Sciences (PIEAS), Nilore, Islamabad, Pakistan; 4 Department of Biotechnology & Informatics, Faculty of life Sciences, Baluchistan University of Information Technology, Engineering and Management Sciences (BUITEMS), Quetta, Baluchistan, Pakistan; Chuo University, JAPAN

## Abstract

The primary goal of cattle genomics is the identification of genome-wide polymorphism associated with economically important traits. The bovine genome sequencing project was completed in 2009. Since then, using massively parallel sequencing technologies, a large number of *Bos taurus* cattle breeds have been resequenced and scanned for genome-wide polymorphisms. As a result, a substantial number of single nucleotide polymorphisms (SNPs) have been discovered across European *Bos taurus* genomes, whereas extremely less number of SNPs are cataloged for *Bos indicus* breeds. In this study, we performed whole-genome resequencing, reference-based mapping, functional annotation and gene enrichment analysis of 20 sires representing eleven important *Bos indicus* (indicine) breeds of Pakistan. The breeds sequenced here include: Sahiwal, Red Sindhi, Tharparkar and Cholistani (tropically adapted dairy and dual purpose breeds), Achai, Bhagnari, Dajal and Lohani (high altitude adapted dual and drought purpose breeds); Dhanni, Hisar Haryana and Gabrali (dairy and light drought purpose breeds). A total of 17.4 billion QC passed reads were produced using BGISEQ-500 next generation sequencing platform to generate 9 to 27-fold genome coverage (average ~16×) for each of the 20 sequenced sires. A total of 67,303,469 SNPs were identified, of which 3,850,365 were found novel and 1,083,842 insertions-deletions (InDels) were detected across the whole sequenced genomes (491,247 novel). Comparative analysis using coding region SNPs revealed a close relationship between the best milking indicine breeds; Red Sindhi and Sahiwal. On the other hand, Bhagnari and Tharparkar being popular for their adaptation to dry and extremely hot climates were found to share the highest number of SNPs. Functional annotation identified a total of 3,194 high-impact (disruptive) SNPs and 745 disruptive InDels (in 275 genes) that may possibly affect economically important dairy and beef traits. Functional enrichment analysis was performed and revealed that high or moderate impact variants in wingless-related integration site (Wnt) and vascular smooth muscle contraction (VSMC) signaling pathways were significantly over-represented in tropically adapted heat tolerant Pakistani-indicine breeds. On the other hand, vascular endothelial growth factor (VEGF) and hypoxia-inducible factor 1 (HIF-1) signaling pathways were found over-represented in highland adapted Pakistani-indicine breeds. Similarly, the ECM-receptor interaction and Jak-STAT signaling pathway were significantly enriched in dairy and beef purpose Pakistani-indicine cattle breeds. The Toll-like receptor signaling pathway was significantly enriched in most of the Pakistani-indicine cattle. Therefore, this study provides baseline data for further research to investigate the molecular mechanisms of major traits and to develop potential genomic markers associated with economically important breeding traits, particularly in indicine cattle.

## Introduction

Livestock contributes 40% of the worldwide estimation of agricultural yield [1]. It provides employment to nearly 1.3 billion people worldwide and directly helps the livings of 0.6 billion farmers in the developing countries [2]. Domestic cattle plays important role in agricultural economy of developing countries, its contribution goes beyond the direct production of milk and meat to skins, fiber, fertilizer and fuel production [3, 4].

Based on a number of phenotypic differences, nearly 800 domestic cattle breeds are divided into two main types, regarded as two subspecies of a species, *Bos taurus* (taurine) and *Bos indicus* (indicine) [5]. Along with many other reported differences between taurine and indicine cattle, the presence of a hump over the shoulder in indicine cattle is a prominent phenotypic difference [6]. More heat tolerance, significantly less susceptibility to ticks, low maintenance cost and resistance to gastrointestinal parasites are the key physiological strengths of indicine over taurine [7]. These physiological advantages enable indicine to grow in tropical and subtropical regions, such as Africa, South-east Asia, Brazil, northern Australia, southern China, and parts of the United States. Whereas, taurine with comparatively higher metabolic rate and nutrient requirements are mostly populated in more developed European countries, north America and Australia [8]. On the other hand, population studies using genomic SNP data showed at least three major geographical divisions of cattle; European, Indian, and African cattle [9, 10].

In Pakistan and India, there are 35 recognized zebu breeds [11]. Cattle in Pakistan is the second largest livestock species after goat, with 42.8 million estimated heads, making nearly 3.2% of the world’s total cattle population [12, 13]. Pakistan is the fourth largest milk producer, recorded 40 million tons in 2011–2012 [1]. There are many indigenous breeds of indicine in Pakistan (please see S1 File for the introduction of Pakistani-indicine breeds). Most zebu breeds from Pakistan are developed as draught cattle [11], whereas Sahiwal and Red Sindhi are specialized dairy cattle. On the other hand Tharparkar, Achai, Gabrali and Cholistani are dual-purpose cattle breeds.

Being the key farm animal that provides major source of essential nutrients and because of their phylogenetic position, representing *Ruminantia*, bovine genome was among the first mammalian genomes sequenced [14]. The bovine genome sequencing consortium presented the bovine reference genomes, UMD and Btau [15, 16], The reference genomes were assembled from shotgun and bacterial artificial chromosome (BAC) capillary sequencing data of an inbred Hereford cow and her sire [17].

Gibbs and his team studied the HapMap Consortium of different cattle populations and provided the foundation for understanding the cattle genetic diversity by scanning 37,000 probes (SNPs) in 497 cattle from 19 phenotypically different and geographically distant breeds [14, 17, 18]. Since then, owing to recent advances in sequencing technologies (next generation technologies) and the availability of bovine reference genome, whole genomes of many cattle breeds from different geographical regions have been resequenced to identify substantial number of SNPs, insertion/deletion (InDels), copy number variations (CNVs) and large structural variations [19–29].

All cattle whole genome resequencing efforts for assessing genomic variations such as the 1000 bull genome project, the international bovine genome sequencing and HapMap projects have catalogued sufficient number of SNPs and small InDels in publicly accessible dbSNP database [17, 18, 30, 31].

High density SNP arrays have been constructed for cattle genome wide association (GWAS) and genotyping studies using the available SNPs in dbSNP databases [32, 33]. For domestic animals, these tools can contribute in understanding the genetic basis of complex agricultural traits and in improving genomic selection methods for enhanced animal production [34, 35].

As of February 2017, only 4.38 million SNPs out of total 99.71 million across the whole bovine genome have been deposited for the indicine breeds in dbSNP database (http://www.ncbi.nlm.nih.gov/snp/), suggesting that more than 96% SNPs are published for taurine breeds. Furthermore, currently available SNP arrays for genotyping, GWAS and genomic selection are constructed from the available dbSNP data, hence they are found biased towards taurine breeds, limiting genome-wide studies of indicine breeds [36]. This bias significantly interrupts the estimates gained from the data [37–39]. However, to overcome the taurine preference, the GeneSeek Inc. (Lincoln, NE) has recently introduced low- and medium-density markers specific arrays for indicine breeds, but these still suffer from ascertainment bias [40, 41]. To overcome taurine preferences, additional whole genome resequencing studies of indicine breeds from geographical different regions are needed to discover and deposit more indicine SNPs [42–44]. Keeping in view the insufficient genomics work done on indicine breeds, scientists have recently released the whole genome sequences of different indicine breeds, which include Nellore [45], Brahman [9], Gir [35], many African breeds [46], three indicine breeds from Brazil [29], and recently resequenced three indicine breeds of Indian origin [47] to fill in the gap. Nonetheless, there is an urgent need to discover substantial number of SNPs across many indicine breeds, belonging to diverse geographical regions, to build new high density SNP-array for unbiased genome wide cattle genotyping and subsequent cattle genomic selection for economically important traits [35].

In this study, we report for the first time the genome characterization of eleven indigenous Pakistani cattle breeds that represent the cattle diversity of the region. Sahiwal is the most milking zebu from dry tropical region of Punjab, Pakistan which shows tolerance to high temperature and resist multiple infectious diseases [48, 49]. Red Sindhi is a tropically adopted and is the second highest milking breed of present day Pakistan [50]; Bhagnari, Dajal, Dhanni and Lohani are the major draught breeds found in hot, mountainous and dry environment of Sibi, Dera Ghazi Khan, Attock and Loralai, respectively [51]. Achai and Gabrali are dairy and light draught breeds adapted to high altitude habitat of Khyber Pakhtunkhwa (KP) and, both are small to medium-sized animals. Tharparkar and Cholistani are fairly good milk producers dual-purpose breeds, and are adopted to extremely hot and dry climate of Tharparkar (Sindh province) and Cholistan (Rahimyar Khan, Bahawalpur and Bahawalnagar districts). This study provides a valuable resource for further investigations of the genetic mechanisms underlying traits of interest in indicine cattle.

## Materials and methods

### Ethics statement

Blood samples for cattle whole-genome resequencing were collected with the consent of the owners of cattle used in this study. The research ethics committee of the National Institute for Biotechnology and Genetic Engineering (NIBGE), Faisalabad, Pakistan has approved the protocols and all animal procedures used for the collection of blood samples from cattle.

### Sampling and DNA fragmentation

Whole-blood samples (10 mL) were collected from two Achai, three Bhagnari, two Cholistani, two Dhanni, one Dajal, two Gabrali, two Hisar Haryana, two Sahiwal, two Tharparkar, one Red Sindhi and one Lohani proven bulls. Next generation sequencing (NGS) pair-end reads were generated using BGISEQ-500 sequencing platform. Genomic DNA (gDNA) was isolated from each sample using FavorPrep gDNA extraction kit (Favorgen Biotech Corporation Ping-Tung AgriBiotech Park, Taiwan) according to the manufacturer’s protocol. The quantity and quality of each recovered gDNA sample were evaluated using agarose gel electrophoresis scans of fluorescently tagged (ethidium bromide) gDNA, spectrophotometer absorbance values, and by measuring double-stranded DNA concentration using qubit dsDNA high sensitivity assay (Molecular probe, Life Technologies). All high quality recovered gDNA samples were randomly fragmented in a separate microtube by ultra-sonication using Covaris S2 (Covaris, USA). Sheared gDNA fragments ranging 500–800 bp in size, were gel purified and for each of the sample, the average fragment size was evaluated using Agilent bio-analyzer 2,100 (Agilent Technologies, USA).

### Library construction

BGISEQ-500 libraries were constructed using slight modification in the Illumina libraries construction method adopted from the previously reported single tube “BEST” procedure, mainly pursuing Carøe et al [52]. To reduce self-ligation, a TrueSeq end repair kit (Illumina) was utilized to generate blunt-ended gDNA fragments by a combination of exonuclease activity and fill-in reaction, prior to advance for a cleanup step using amPure xp beads (Beckman Coulter Genomics, USA). The blunt-ended gDNA fragments were then prepared for ligation to the sequencing adaptor by adding an extra A- base to the 3′ end. Following A-tailing, gDNA fragments were ligated to the T-indexed BGISeq-500 adapters. The ligation products (gDNA libraries) were then recovered by AMPure XP Beads. The desired size-range of all gDNA libraries were excised using gel electrophoresis, and the size-selected gDNA libraries were then extracted from the gel and purified with the help of spin column.

After the final best fill-in step in gDNA library construction, all gDNA libraries were mixed with Qiagen PB binding buffer (1:5 volume) and cleaned by employing Monarch DNA clean-up columns (New England Biolabs, Massachusetts, USA). For washing, size-selected gDNA libraries were first incubated at 37◦C for 5-minutes in 750 μL PE buffer (Qiagen) and finally purified gDNA libraries were eluted using 40 μL Qiagen EB.

For all libraries the number of indexed PCR cycles were determined in qPCR-quantification by using common primer, BGI-forward primer and one of the eight index ligated reverse primers (using the Agilent MX3005 qPCR machine). All libraries were subsequently amplified in 9 to 17 index PCR cycles by using common primer, the index ligated reverse primers, and BGI-forward primer. Each purified BGISEQ-500 library was subjected to an additional refinement step to remove any remaining impurities and primer dimers. All amplified libraries were sent to BGI sequencing facility for independent circularization and high throughput sequencing on BGISEQ -500 NGS platform. Sequences are available from CNGB Nucleotide Sequence Archive (CNSA) with the Bioproject accession number CNP0000189.

### Mapping, InDel realignment, reads de-duplication, and BQSR

After verifying read qualities using FastQC, SOAPduke filter is used to remove adapter sequences, low-quality base calls and N’s from both ends of sequenced reads, and finally clean reads were subjected to reference genome mapping. Clean pair-end reads were mapped to the bovine reference genome assembly UMD 3.1 [16] using the BWA algorithm with default settings [53]. We used open-source software packages for downstream processing and variant calling.

The PCR duplicates were removed using the Picard (v. 1.105) “MarkDuplicate” command line utility (*Picard Tools—By Broad Institute*. https://broadinstitute.github.io/picard/) for bam files. Mapped reads were then analyzed to resolve the mapping issues emerged from the presence of small InDels at the sequenced reads ends by using “RealignerTargetCreator” and “InDelRealigner” command line tools of the Genome analysis toolkit v. 3.3.0 (GATK) [54, 55]. After fixing the alignment issue, the PCR duplicates were removed using the Picard “MarkDuplicate” command line utility. The GATK “BaseRecalibrator” and “PrintReads” command-line tools were used for base quality score recalibration (BQSR) to resolve inordinately high or low base quality scores estimations caused by various systematic and technical sequencing errors [54]. Prior to call variants, indexes were generated for reference and bam files using Samtools, Picard and BWA [53, 56].

### Variant calling, VQSR, and filtering

The GATK command-line tool “HaplotypeCaller” was adopted to call SNPs together with InDels through local *de-novo* assembly of haplotypes in the regions showing deviation [54]. All SNPs and InDels were discovered as differences with the reference UMD 3.1 genome sequence [16]. Variant quality score recalibration (VQSR) was carried out by using the "Variant Recalibrator" and "ApplyRecalibration" GATK command-line tools, to generate a well-calibrated likelihood—for each variant location in a call set—that a SNP is a real hereditary variant against a sequencing or data processing artifact. To remove possible false positive calls and filter variants, the GATK “Variant Filtration” argument and bcf tools filter was adopted using following options: (1) variants were removed having less than 10% of the over-all mean read depth or greater than the mean read depth + three times the standard deviation; (2) SNPs and InDels were removed with total quality less or equal to 20 or less than mean_QUAL, or even greater than mean_QUAL + 2/3 of the QUAL standard deviation; (3) SNPs within 5 base pair of another SNP or an InDel were removed; (4) from the cluster of InDels separated by 10 base pairs or few, only one InDel is retained and the rest are removed; (5) SNPs or InDels with fewer than one reverse or forward allele read were also excluded; (6) SNPs and InDels with phred-scaled P-value using Fisher’s exact test (FS) > 60 and > 200, respectively, were filtered-out, since greater FS score represents strand bias between the two DNA strands for the alternative or reference allele, which are signs of false-positive calls, and (7) SNPs and InDels discovered in unplaced contigs were also eliminated from the downstream analysis [35, 46].

### Variant functional annotation and GO enrichment

After variant filtration, SNPs and InDels were functionally annotated using SnpEff_4.3 [57] to attribute each variant by a functional class and offer various fields of information for the coding variants to identify the affected transcript and protein. The SnpEff databases UMD3.1.87 were used during the course of functional annotation. The SnpEff functional class vocabulary assigned to both SNPs and InDels were exonic, intronic, intergenic, splice site acceptor, splice site donor, splice site region, downstream, upstream, UTR 3 prime and UTR 5 prime. Functional classes exclusively used for InDels were conserved in-frame deletion and insertion, disruptive in-frame deletion and insertion, whereas stop lost, stop retained, initiator codon and synonymous were only assigned to SNPs.

PANTHER enrichment analysis was conducted to identify over-represented biological process categories using all 265 known genes harboring high impact non-synonymous variants in all resequenced samples in this study (GO Ontology database Released 2018-04-04) [58]. Binomial test with Bonferroni correction for multiple testing was executed and a P-value of less than 0.01 was chosen as an inclusion criterion for functional categories.

## Results and discussion

### Sequencing and read alignment

**Fig 1:**
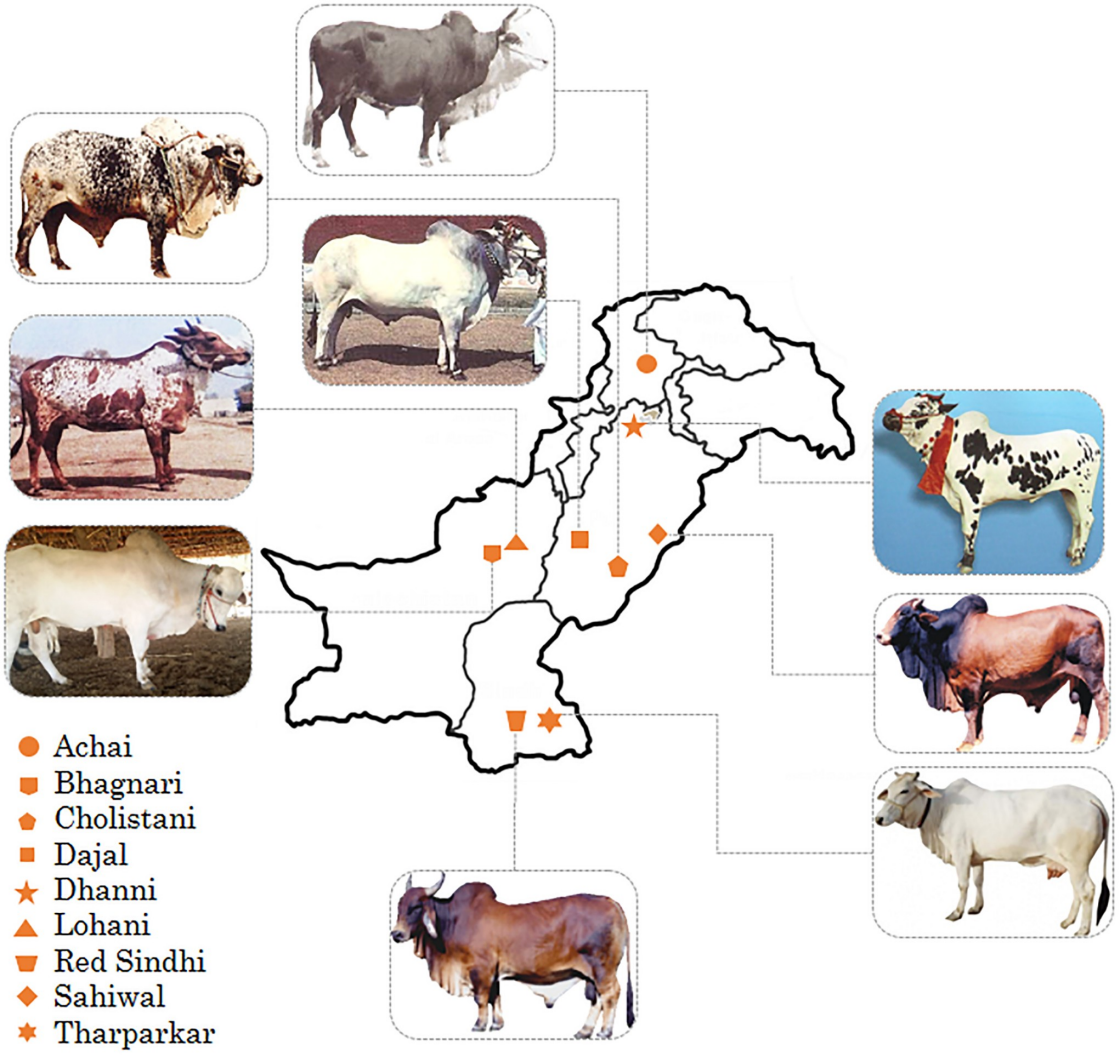
Geographical distribution of indicine breeds in Pakistan.

Individual genomes of 20 native Pakistani cattle from 11 different *B*. *indicus* breeds were sequenced to an average of ~16× coverage each (9× to 27× coverage range). To facilitate comparisons with African cattle, the sequenced cattle data were jointly analyzed with publicly available whole genome sequenced data of African cattle breeds (Boran, Ogaden, Kenana, Ankole, and N’Dama). The *B*. *indicus* breeds from Pakistan comprise of dairy breeds (Sahiwal and Red Sindhi), draught breeds (Bhagnari, Dajal, Dhanni, and Lohani) and dual purpose breeds (Achai, Gabrali, Tharparkar, and Cholistani) (Fig 1). In total, 17.4 billion QC passed filtered reads were generated. Using BWA [53], filtered reads were mapped to the most recent available taurine reference genome UMD 3.1 with an average mapping rate of 94.19%. The clean sequenced data covered 98.56% and 91.53% of the total reference genome with at least one and five or more reads, respectively (Table 1). The mapping results are found analogous to the mapping rates stated in previous studies using the BGISEQ-500 system [59, 60]. Compared with earlier genome resequencing studies in cattle [22, 23, 45, 46, 61], the depth of coverage is sufficient for detecting high-confidence variants.

**Table 1:** Summary of sequencing data.

Breed	Sample ID	Total reads	Aligned reads rate (%)	Average read depth	% Genome covered with at least	
					3 read	4 read
**Cholistani**	3702-C	913,370,542	878,299,353 (96.16%)	17.5	98.6	98.1
**Cholistani**	6314-C	958,697,262	932,760,331 (97.29%)	16.5	89.7	84.7
**Achai**	64MCA	656,833,134	637,879,905 (97.11%)	12.0	96.8	94.8
**Achai**	7CA	787,010,328	763,311,294 (96.99%)	14.3	98.0	97.1
**Bhagnari**	BN_18	363,040,368	332,082,134 (91.47%)	6.3	86.3	75.8
**Bhagnari**	BN_20	965,559,976	939,885,288 (97.34%)	17.7	98.1	97.7
**Bhagnari**	BN_23	754,633,704	733,922,138 (97.26%)	13.9	97.5	96.2
**Dajal**	DajalC	490,209,280	477,845,041 (97.48%)	9.0	95.2	91.1
**Dhanni**	DH287	890,256,004	863,001,339 (96.94%)	16.2	98.6	98.1
**Dhanni**	DH-363	907,603,906	883,526,944 (97.35%)	16.5	95.9	94.1
**Gabrali**	G-27	956,674,298	931,578,709 (97.38%)	17.5	98.4	97.9
**Gabrali**	G-3	491,782,144	481,701,083 (97.95%)	9.1	95.4	91.5
**Hisar Haryana**	HH-44	650,179,266	632,825,348 (97.33%)	11.9	97.1	95.2
**Hisar Haryana**	HH-46	759,783,444	737,015,449 (97.00%)	13.8	97.8	96.5
**Sahiwal**	Zaibee	907,603,906	802,265,635 (88.4%)	15.0	97.8	96.7
**Tharparkar**	TH-138	912,848,966	888,545,930 (97.34%)	13.7	98.4	97.3
**Tharparkar**	TH-158	902,603,906	877,526,944 (97.35%)	16.7	98.5	97.9
**Lohani**	Lohani-18	1,419,121,650	1374,888,332 (96.88%)	25.8	98.9	97.9
**Red Sindhi**	RS_303	1,340,604,416	1297,472,741 (96.78%)	24.4	98.2	92.8
**Sahiwal**	Sunny	1,491,407,896	1,452,737,015 (97.41%)	27.3	98.8	97.7

### Variant identification: (SNPs) and (InDels)

**Fig 2:**
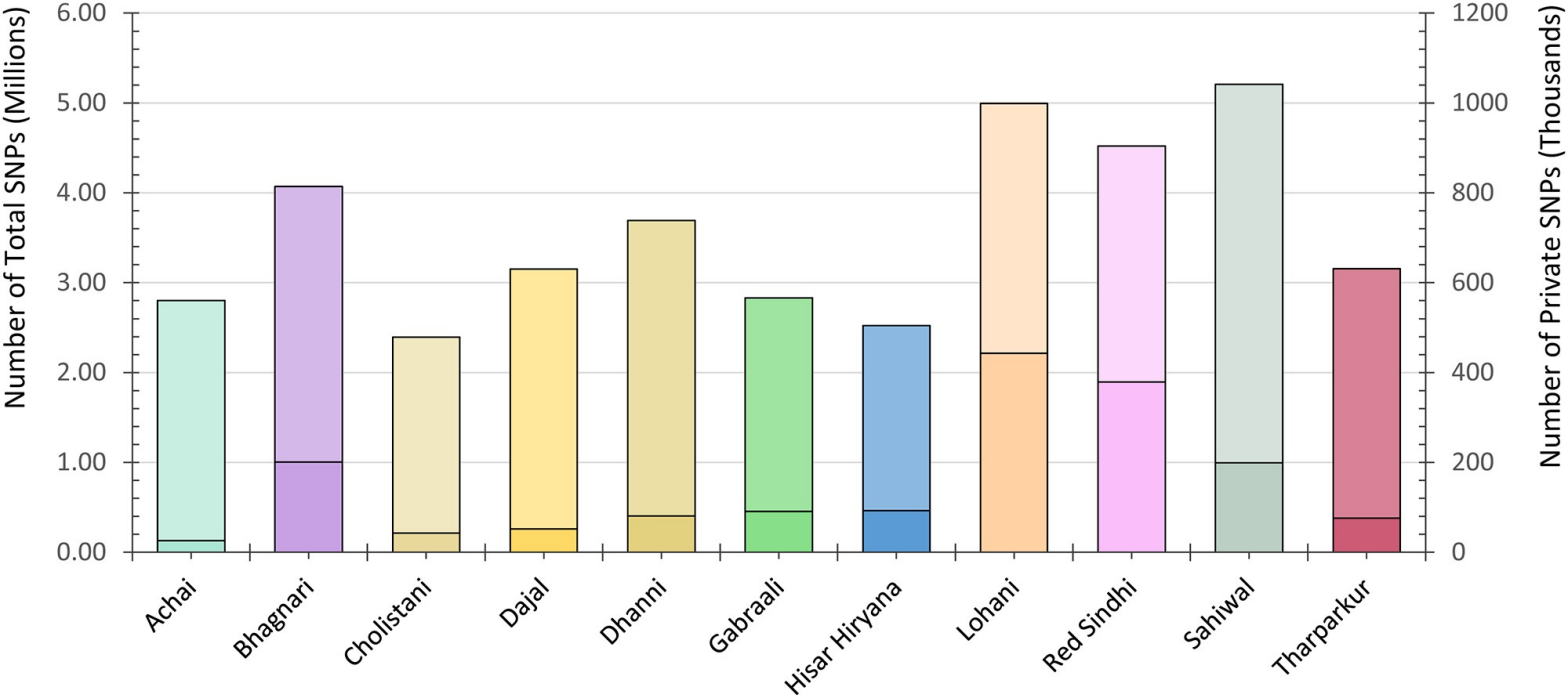
Total and private SNPs count in different breeds of Pakistan.

In this study, a total of ~67.3 million SNPs and ~1.08 million InDels were finally retained across all sequenced genomes, with an overall average change rate of 1 in 827 base pairs (Fig 2 and S1 Table). All SNPs are deposited in European variation archive (EVA) EMBL-EBI under project identifier PRJEB29251, with analysis accession number from "ERZ776599" to "ERZ776619". A full list of analysis accession numbers for all 20 VCFs is provided in S2 Table.

The 3,850,365 SNPs and 491,247 InDels were found novel when compared against dbSNP build 148. The proportion of novel SNPs is lower than reported in previous cattle resequencing studies, such as in Fleckvieh, Holstein, Black Angus, Kuchinoshima Ushi, Boran, Ogaden, Kenana, Ankole, N’Dama and Chikso breeds [22–24, 46, 62]. The lower proportion of novel SNPs may be due to the recent SNPs depositions from latest large number of bovine resequencing studies, and may also be accounted by the use of stringent variant filtering standards to minimize false positive variant calls. Compare to SNPs, a higher proportion of novel InDels could in part be because a large number of next generation resequencing experiments in cattle have focused on reporting SNPs rather than InDels. Only 1.6% of the total variations discovered in this study are attributed to InDels. However, the InDels involve nearly 3.8% of the entire variant bases, implying that InDels may be a significant source of both genomic and phenotypic diversity.

Among the total SNPs and InDels, the homozygous and heterozygous ratios were 1:1.3 (32,890,916 verses 34,412,553 SNPs) and 1:1.27 (479,060 versus 604,782 InDels), respectively. To evaluate the quality of identified SNPs, the transition (Ts) versus transversion (Tv) ratio of all detected SNPs was measured and on an average, we found 2.3 Ts versus 1 Tv (Ts: Tv 2.3:1) across the entire resequenced genome of all samples under study. The Ts: Tv ratio value is analogous to the ratios documented in other related studies for cattle and human. [24, 28, 63].

**Fig 3:**
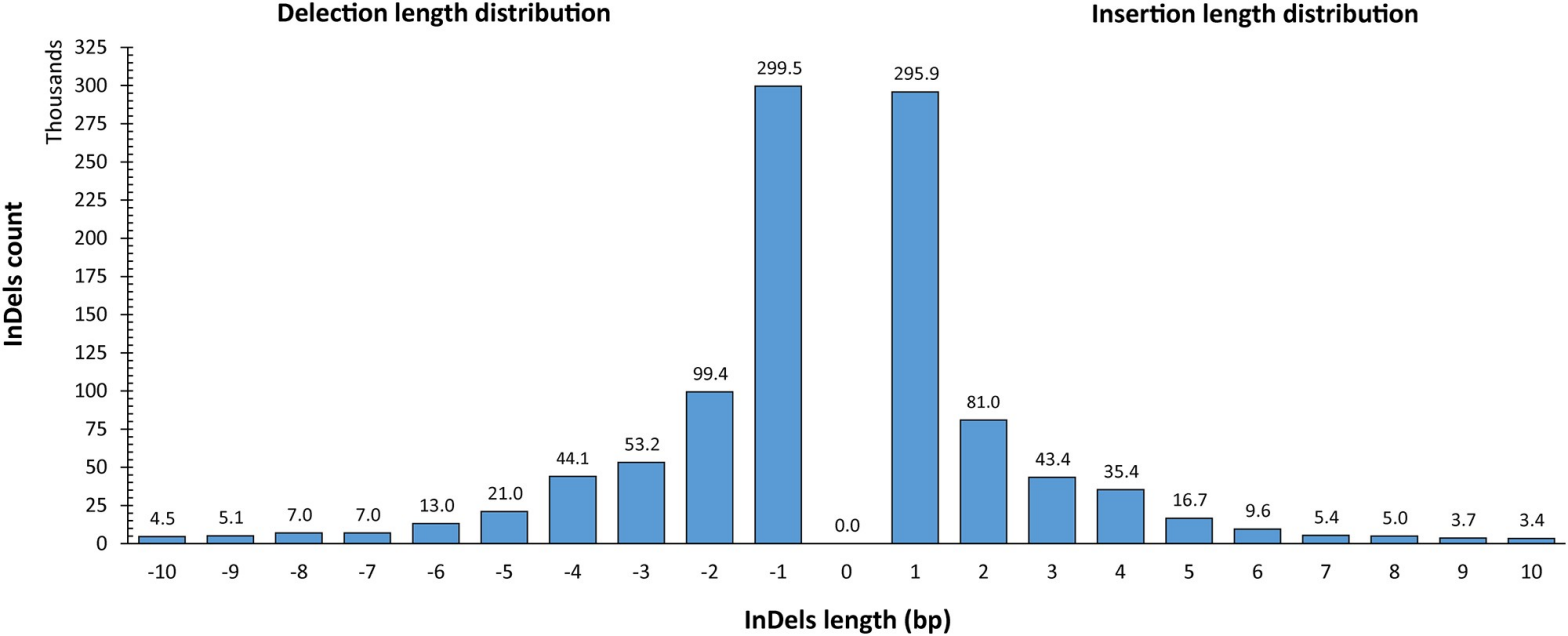
All InDels length distribution.

Among 1,083,842 InDels, 573,528 (52.9%) are deletions. The lengths of InDels ranges from -26 (deletion) to 20 (insertion). However, most of the determined InDels are short: approximately 80.48% of InDels are less than and equal to 3bp (Fig 3) which is comparable to the previous report [22, 24]. The minimum depth for InDels was set to 6 supporting reads, resulting in nearly 98.3% of InDels with at least 10 assisting reads in the final call set. In addition, InDels with very high read depth (mean + 3*Std deviation) were also excluded to minimize possible PCR artifacts. These results collectively show that the sequencing data supports the identified InDels.

### Variant based genomic comparison

**Fig 4:**
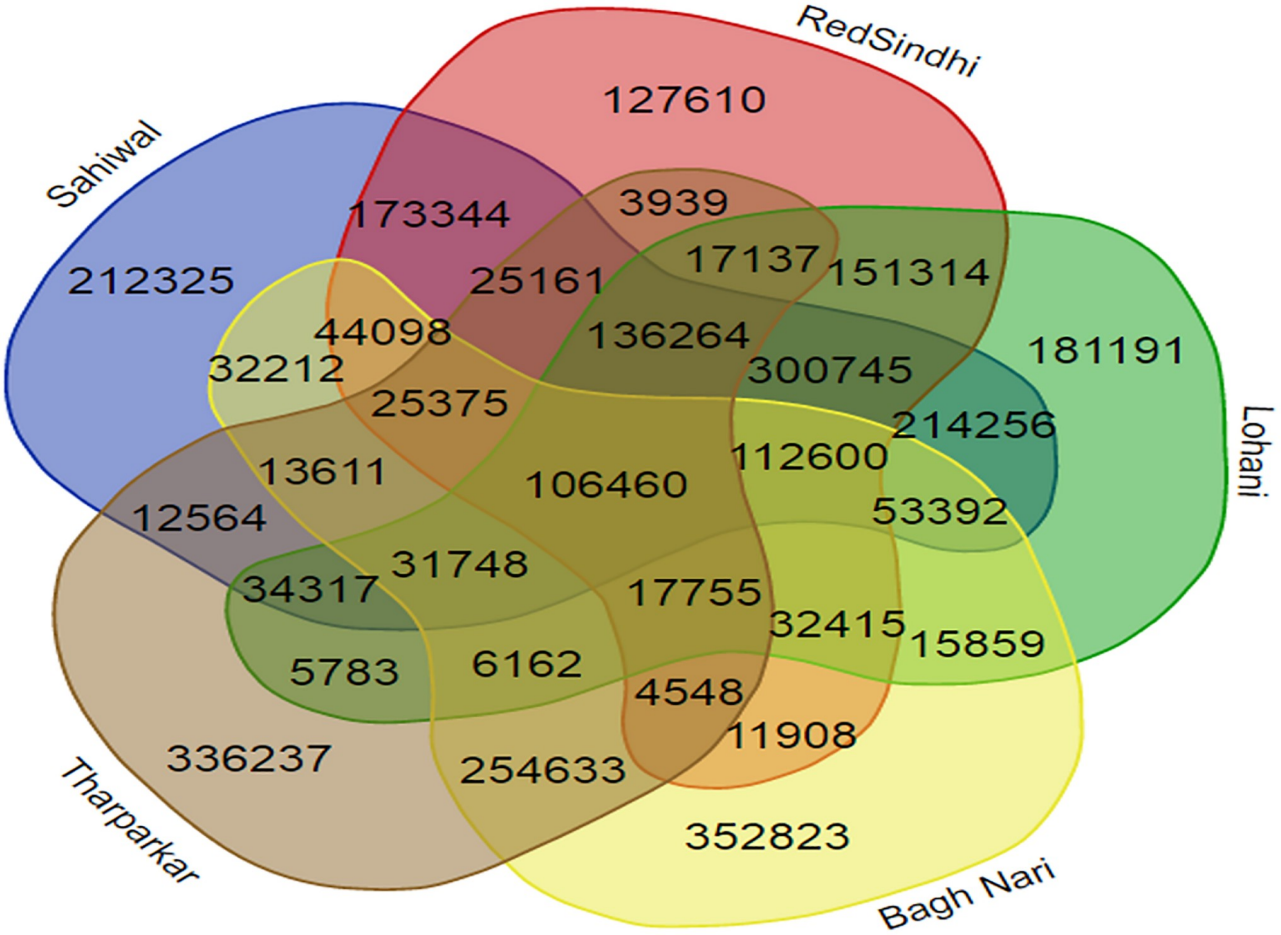
Venn diagram showing coding region SNPs based comparison of five important Pakistani indicine cattle breeds.

In this study, a large number of SNPs were found being shared among all sequenced breeds. We compared genic and regulatory (1,000 bp up and down stream) region SNPs among top five important indicine breed of Pakistan; Sahiwal, Red Sindhi, Bhagnari, Lohani and Tharparkar. A total of 106,460 (1.7%) coding region SNPs were common to all five breeds. While breed specific SNPs (Private SNPs) represented 32.6% in Tharparkar, 31.6% in Bhagnari, 13.9% in Sahiwal, 12.8% in Lohani and 9.9% in Red Sindhi (Fig 4).

Breed specific SNPs could possibly be helpful in further research concerning breed characterization, whereas the extent of relations between breeds may be determined by the number of shared SNPs between them. Although Bhagnari is a heavy drought breed mostly raised for beef production, while Tharparkar is a dual purpose breed and fairly good milk producer, we identified larger number of shared coding SNPs common to Bhagnari and Tharparkar (254,633 SNPs) may be due to fact that both are adopted to extreme hot and dry tropical regions (Sibi Baluchistan and Thar Sindh) and share similar grey to white coat color—than those common to Sahiwal and Bhagnari (32,212 SNPs), or to Red Sindhi and Bhagnari (11,908 SNPs) or even to Lohani and Bhagnari (15,859 SNPs). Similarly, we found that Red Sindhi, being the second best milking indicine breed shares the highest number of coding SNPs with the best milk producer Sahiwal breed (173,344 SNPs), both breeds have also been reported to withstand heat and resist several infectious diseases [64].

### SNPs functional annotation

The final variant set was extensively investigated to functionally annotate every single SNP and InDel with their particular attributes by using SnpEff_4.3 [57]. Of the total SNPs discovered in this study, 48,228,045 (71.7%) are found in between the genes. A total of 550,845 (0.818%) SNPs are located in a thousand base pairs (bp) upstream region of all genes, whereas 598,530 (0.889%) are positioned in a thousand bp downstream regions from the genes set. The remaining 21,541,768 (27%) SNPs are found in coding regions (Table 2). More functional effects were observed compared to the total number of SNPs and InDels used for variant functional annotation, because some variants have more than one functional effects, possibly they are part of different isoforms or even overlapping genes.

**Table 2:** Annotation of SNPs detected in all sequenced Pakistani *Bos indicus* breeds.

SNPs	Count	Percentage
INTERGENIC	48228045	**67.56%**
INTRONIC	20886071	**29.26%**
DOWNSTREAM	598530	**0.84%**
UPSTREAM	550845	**0.77%**
EXONIC	426173	**0.60%**
SILENT	255296	**0.36%**
MISSENSE	155251	**0.22%**
UTR_3_PRIME	153614	**0.22%**
SPLICESITEVARIANT (SP)	53841	**0.08%**
SPLICE_SITE_REGION	49505	**0.07%**
UTR_5_PRIME	23951	**0.03%**
SPLICE_SITE_ACCEPTOR	1299	**0.00%**
SPLICE_SITE_DONOR	1155	**0.00%**
STOPGAIN (SG)	1132	**0.00%**
NONSENSE	1132	**0.00%**
START_LOST (STL)	104	**0.00%**
STOPLOSS (SL)	71	**0.00%**
KNOWN	68347188	
NOVEL	3850365	
**TOTAL**	**72197553**	

Large majority of the genic SNPs are identified in the intronic region (20,886,071; 26%). A total of 157,690 (0.234%) genic SNPs are found to introduce non-synonymous (nsySNPs) mutations; of which 155,251 (0.23%) SNPs were predicted to introduce amino acid substitutions (missense) and 1132 (0.00168%) SNPs were detected to change amino-acids specifying codons into stop codons (nonsense), whereas many other nsySNPs, such as stop-gain, start-loss and stop-loss (1132; 104 and 71, respectively) were also detected in this study. Such nsySNPs may influence phenotypic variation in economically important traits in cattle. The identified variants were annotated with the latest available dbSNP (Build 148), revealing 3,850,365 novel SNPs. Following annotation, we further investigated nsySNPs using SnpEff_4.3 and identified 3194 high (disruptive), 162,740 moderate and 316,266 low impact SNPs in all sequenced *B*. *indicus* breeds from Pakistan (S3 Table).

### InDels functional annotation

InDels events were also searched in all sequenced samples and detected 1,083,842 InDels in total, 592,595 (54.74%) of which were found in the dbSNP database, whereas the remaining 491,247 (45.32%) are novel. The functional annotation using SnpEff_4.3 found a total of 769,078 (67.14%) and 349,596 (30.52%) InDels in intergenic and intronic regions, respectively (Table 3).

**Table 3:** Annotation of InDels detected in all sequenced Pakistani *Bos indicus* breeds.

InDels	Count	Percentage
INTERGENIC	769078	**67.14%**
INTRONIC	349596	**30.52%**
DOWNSTREAM	11370	**0.99%**
UPSTREAM	9316	**0.81%**
UTR_3_PRIME	2982	**0.26%**
EXONIC	1079	**0.09%**
SPLICE_SITE_REGION	850	**0.07%**
FRAMESHIFT_VARIANT	602	**0.05%**
UTR_5_PRIME	277	**0.02%**
SPLICE_SITE_ACCEPTOR	101	**0.01%**
DISRUPTIVE_INFRAME_DELETION	92	**0.01%**
DISRUPTIVE_INFRAME_INSERTION	56	**0.00%**
CONSERVATIVE_INFRAME_INSERTION	53	**0.00%**
CONSERVATIVE_INFRAME_DELETION	50	**0.00%**
SPLICE_SITE_DONOR	42	**0.00%**
STOP_GAINED (SG)	11	**0.00%**
START_LOST (STL)	9	**0.00%**
STOP_LOST (SL)	2	**0.00%**
KNOWN	592595	
NOVEL	491247	
**TOTAL**	**1083842**	

**Fig 5:**
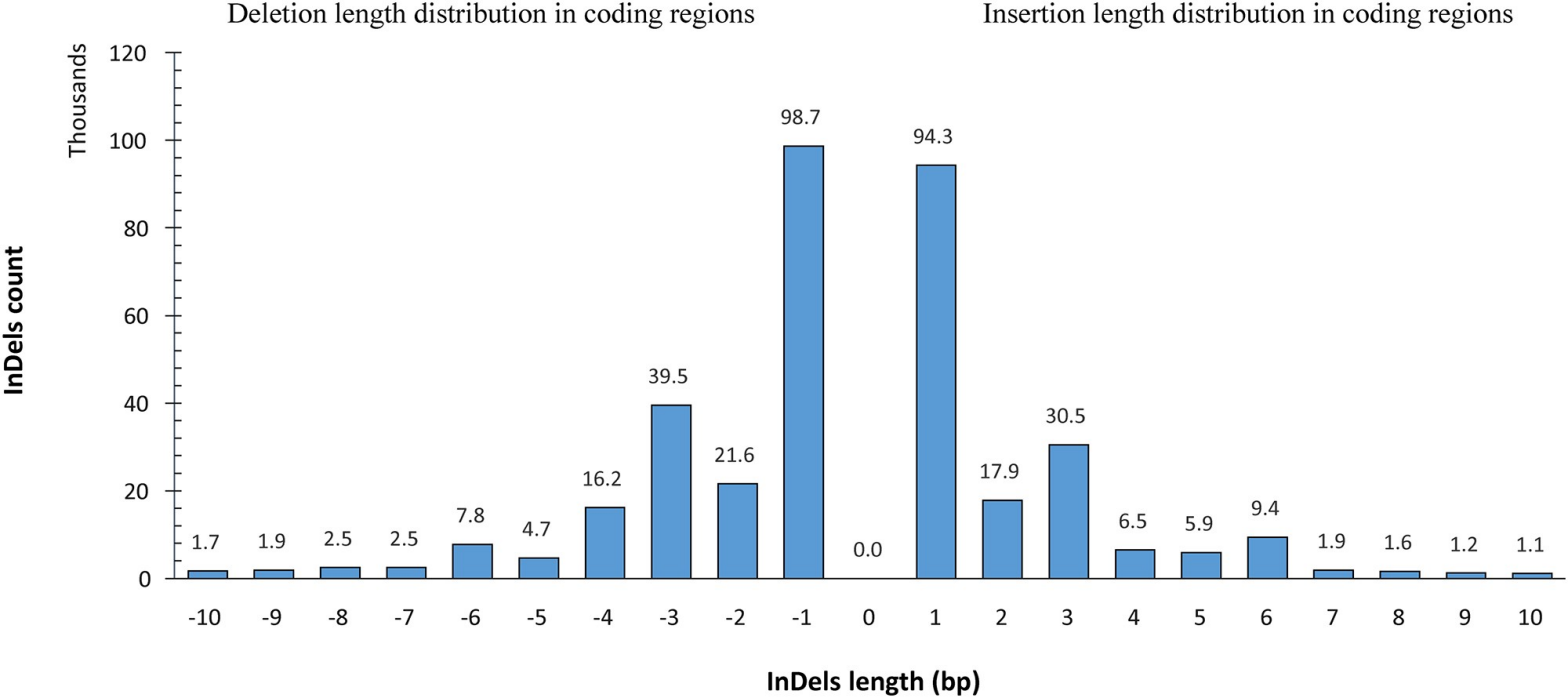
InDels length distribution found in coding regions.

Moreover, 9,316 (0.85%) 11,370 (0.99%) InDels are located within the 1000 base pair upstream and downstream genic region, respectively. Following annotation, we investigated the length distribution of all discovered InDels and InDels in the coding regions. As shown in Fig 5, a total of 34,430 (8.87%) and 8,082 (2.08%) InDels found in the coding region are 3-bp and 6-bp, respectively as has been reported in the human population [65]. Such hereditary variants are likely to be more endured and less selected than those causing frame-shift mutations. Only 745 (0.07%) high impact non-synonymous (disruptive) InDels were detected in all 20 sequenced samples, of which 604 (0.06%) were discovered to cause frame-shift in 275 genes.

### Functional enrichment analysis

Generally, indicine cattle can withstand high temperature compared to taurine breeds. In Pakistan, indicine cattle inhabit hot regions of Punjab, Sindh and Baluchistan provinces, and have evolved to adapt harsh environmental conditions, such as high temperature and solar radiation, infectious diseases, and poor diet. These environmental conditions dominate across most of the indicine populated regions of Pakistan. Through comparisons of the genomes of indicine breeds inhabiting extreme environments in Pakistan with the reference taurine (Herefords breed) genome from contrasting environments, we aimed to identify the functional gene ontology (GO) biological processes and KEGG signaling pathways responsible for the adaptations of indicine breeds to hot, humid and plateau environments of Pakistan. To investigate this; all identified SNPs are functionally annotated by SnpEff_4.3 to predict high (triggering loss/gain of function or protein truncation) and moderate (a non-disruptive variant that could change protein effectiveness) impact variants that could possibly alter economically important protein coding genes. Such genetic variants of high or moderate severity provide a useful resource for further studies to phenotypically differentiate taurine and indicine breeds. Our analysis showed a total of 462 genes (256 known genes) in all Pakistani-indicine population harboring at least one SNP of high or moderate severity. The GO enrichment analysis was performed for high or moderate impact SNPs harboring 256 known genes (of the total 462 predicted gene) using PANTHER overrepresentation test (Released 2017-12-05) [58]. The GO enrichment analysis revealed five GO immune responses associated biological processes (GO:0090389~phagosome-lysosome fusion involved in apoptotic cell clearance, GO:0090387~phagolysosome assembly involved in apoptotic cell clearance, GO:0048290~isotype switching to IgA isotypes, GO:0002826~negative regulation of T-helper 1 type immune response, GO:0038172~interleukin-33-mediated signaling pathway). We found four heat tolerance related GO biological processes (GO:0042309~homoiothermy, GO:0021985~neurohypophysis development, GO:0010224~response to UV-B, GO:0033326~cerebrospinal fluid secretion). Four GO biological processes related to signal transduction (GO:0050962~detection of light stimulus involved in sensory perception, GO:0050908~detection of light stimulus involved in visual perception, GO:2000050~regulation of non-canonical Wnt signaling pathway, GO:0035021~negative regulation of Rac protein signal transduction) were found. Two cellular development associated GO biological processes (GO:0048675~axon extension, GO:0032292~peripheral nervous system axon ensheathment) were enriched in all eleven breeds studied, considering a 10%FDR threshold for significance (Table 4).

**Table 4:** Gene ontology (GO) based on known genes harboring high or moderate impact SNPs.

GO biological process complete	Fold Enrichment	P-value
**Immune responses**		
Phagosome-lysosome fusion involved in apoptotic cell clearance (GO:0090389)	82.97	1.20E-02
Phagolysosome assembly involved in apoptotic cell clearance (GO:0090387)	82.97	1.20E-02
Isotype switching to IgA isotypes (GO:0048290)	82.97	1.20E-02
Negative regulation of T-helper 1 type immune response (GO:0002826)	82.97	1.20E-02
Interleukin-33-mediated signaling pathway (GO:0038172)	41.48	2.38E-02
**Heat tolerance**		
Homoisothermy (GO:0042309)	82.97	1.20E-02
Neurohypophysis development (GO:0021985)	82.97	1.20E-02
Response to UV-B (GO:0010224)	11.85	1.27E-02
Cerebrospinal fluid secretion (GO:0033326)	41.48	2.38E-02
**Signaling pathways**		
Detection of light stimulus involved in sensory perception (GO:0050962)	9.76	1.83E-02
Detection of light stimulus involved in visual perception (GO:0050908)	9.76	1.83E-02
Regulation of non-canonical Wnt signaling pathway (GO:2000050)	9.22	2.03E-02
Negative regulation of Rac protein signal transduction (GO:0035021)	20.74	4.71E-02
**Cellular development**		
Exon extension (GO:0048675)	7.32	8.39E-03
Peripheral nervous system axon ensheathment (GO:0032292)	7.93	2.70E-02

In this study, seven KEGG (Kyoto Encyclopedia of Genes and Genomes) pathways were identified as being significantly over-represented (p < 0.05) by DAVID in at least two of the Pakistani indicine breed (Table 5). The Wnt signaling pathway (bta04310) was over-represented in Sahiwal, Tharparkar and Cholistani breeds. The vascular smooth muscle contraction (VSMC) pathway (bta04270) was over-represented in Sahiwal, Red Sindhi, Tharparkar, Bhagnari and Lohani breeds. The vascular endothelial growth factor (VEGF) signaling pathway (bta04370) was over-represented in Bhagnari and Lohani breeds and, hypoxia-inducible factor 1 (HIF-1) signaling pathway (bta04066) was found over-represented in Achai, Bhagnari and Lohani breeds. The extracellular matrix (ECM) receptor interaction pathways (bta04512) was over-represented in Sahiwal, Red Sindhi and Tharparkar breeds. The Janus kinase-signal transducers and activators of transcription (Jak-STAT) pathway (bta04630) was over-represented in Achai, Bhagnari and Cholistani breeds. The Toll-like receptor signaling pathway (bta04620) was over-represented in Red Sindhi, Tharparkar, Achai, Bhagnari and Lohani breeds. For the detailed description of all over-represented KEGG pathways, please see supplementary information **(**S2 File).

**Table 5:** KEGG pathways enriched (FDR<0.10) in Sahiwal, Red Sindhi, Tharparkar, Cholistani, Achai, Bhagnari and Lohani indicine breeds from Pakistan.

	Sahiwal		Red Sindhi		Tharparkar		Cholistani		Achai		Bhagnari		Lohani	
KEGG Pathways	Genes	FDR	Genes	FDR	Genes	FDR	Genes	FDR	Genes	FDR	Genes	FDR	Genes	FDR
**Wnt signaling pathway (bta04310)**	36	9.16E-23	-	-	37	3.04E-31	18	1.02E-09	-	-	-	-	-	-
**Vascular smooth muscle contraction (bta04270)**	39	5.28E-29	31	4.42E-20	23	2.23E-14	-	-	-	-	42	1.91E-38	15	1.09E-07
**VEGF signaling pathway (bta04370)**	-	-	-	-	-	-	-	-	-	-	24	1.04E-21	29	6.82E-37
**HIF-1 signaling pathway (bta04066)**	-	-	-	-	-	-	-	-	15	2.23E-14	27	3.93E-20	30	2.34E-31
**ECM-receptor interaction (bta04512)**	39	3.78E-35	46	1.91E-48	25	2.35E-20	-	-	-	-	-	-	-	-
**Jak-STAT signaling pathway (bta04630)**	-	-	-	-	-	-	26	1.16E-18	10	7.70E-05	34	5.35E-23	-	-
**Toll-like receptor signaling pathway (bta04620)**	-	-	27	7.24E-17	20	6.65E-12	-	-	19	8.58E-21	23	4.65E-14	15	1.94E-08

#### Heat tolerance in Sahiwal, Red Sindhi, Tharparkar and Cholistani cattle

Wnt proteins are specialized secretory proteins that play role in different cellular developmental processes by promoting morphogenesis, differentiation of progenitor-cells into more specific types, cell fate specification, and controlling irregular cell divisions in different tissues and organs. Whereas, the VSMC is a specialized contracting cell required for the regulation of blood flow and pressure in different stress conditions, by adjusting the diameter of the blood vessels. The body’s core internal temperature controlled by skin blood flow is critical during external and internal challenges to thermal homeostasis [66]. The Wnt and VSMC signaling pathways were found significantly over-represented (each by fifty-seven genes) in tropically adapted heat tolerant Sahiwal, Red Sindhi, Tharparkar and Cholistani indicine breeds of Pakistan. Along with other skin blood flow regulating pathways, Kim et al. [46] identified Wnt signaling pathway significantly enriched in thermos-regulating African indicine cattle, and Yang et al. [67] reported the significant enrichment of VSMC pathway by a set of candidate genes in native highland sheep adapted to extremely hot environments. These pathways might explain the capabilities of tropically adapted Pakistani indicine breeds to withstand high temperature at the cellular and physiological levels compared to temperate taurine Hereford breed.

#### High altitude adaptation of Achai, Bhagnari and Lohani breeds

VEGF signaling pathway is a VEGF to VEGFR-2 binding dependent cascade of chemical reactions that plays a crucial role in both pathologic and physiologic formation of blood vessels, called angiogenesis. VEGFR-2 is a major mediator of VEGF driven response in endothelial cells. The VEGFR-2 to VEGF binding initiates various signaling transduction pathways leading to endothelial cell proliferation and subsequent migration to ensure their survival and vascular permeability. Whereas, the HIF-1 is a transcription factor that functions as a master gene regulator of numerous hypoxia-inducible protein-coding genes under hypoxic condition. The HIF-1 targeted genes encode proteins that facilitate oxygen supply in response to oxygen depletion. Many human and rodent genes from VEGF and HIF-1 pathways are found associated with the regulation of cardiovascular system under hypoxic conditions [68–73]. Among the total genes harboring high or moderate impact variants in highland adapted Pakistani-indicine breeds (Achai, Bhagnari and Lohani), thirty-one were located in VEGF signaling pathway; thirty-four were found in HIF-1 signaling pathway, which plays an essential part in the regulation of cellular responses to depleted oxygen condition [74]; and forty-seven discovered in VSMC signaling pathway, which plays a key role in the delivery of blood oxygen by regulating the vasodilation [67]. Tuder et al. [75] reported enhanced mRNA expressions for VEGF and VEGF-receptors in the lungs of *ex-vivo* low oxygen treated rats. In 2016, Yang and colleague [67] reported significant enrichment of VEGF, HIF-1, and VSMC signaling pathways (along with others) by a set of newly discovered genes associated with hypoxia in native sheep, rearing at high altitudes. In response to high altitude hypoxia, a higher expression for HIF‐1 and its regulated genes along with VEGF, glucose transporter-1 and hexokinase-2 were also reported in highland cattle population inhabiting the trans-Himalayan region [76]. The results indicated that responses to hypoxia, development of new blood vessels and their dilatation (angiogenesis and vasodilatation) in stress conditions are the important factors that allow highland Pakistani-indicine cattle to cope with the oxygen depletion at higher altitudes (Please see S1 File for all identified genes enriching KEGG pathways in Pakistani indicine breeds).

#### The impact of human selection on dairy and beef traits enrichment in Sahiwal, Red Sindhi and Tharparkar cattle

The ECM is a mixture of complex structural and functional macromolecules that maintain the structure and function of cells and tissues, with an essential role in the morphogenesis of tissue and organ. Its interaction with the transmembrane cellular molecules can directly or indirectly control the cellular migration, adhesion, proliferation, apoptosis and differentiation. The ECM-receptor interaction pathway was significantly enriched in Pakistani dairy cattle breeds (Sahiwal, Red Sindhi and Tharparkar) by sixty-five genes harboring high or moderate impact variants. Gao et al. [77] reported transcriptional regulation of ECM-receptor interaction pathway during lactation period in Holstein cows and was found over-represented by a set of candidate genes in Brazilian dairy crossbred Girolando cattle (Gyr x Holstein) and a Brazilian dual-purpose Guzerat breed [29].

In mammals, the Jak-STAT pathway is the major signaling mechanism for a wide array of cytokines and growth factors that transduces a multitude of signals and control the expression of target genes for the development and homeostasis in animals, from humans to flies. In this study, the Jak-STAT signaling pathway was found significantly over-represented in meat purpose Pakistani-indicine breeds (Achai, Bhagnari, and Cholistani) by a total of fifty high or moderate impact variation harboring genes. Doran, et al. [78] also found Jak-STAT and other related signaling pathways associated with various aspects of bovine carcass performances.

#### The adaptation of Pakistani-indicine cattle to infectious diseases

Toll-like receptors (TLRs) are specific families of pattern recognition receptors, responsible for detecting microbial pathogens and produce innate immune responses to infectious microbes. Mammalian TLRs are membrane-bound receptors, expressed on macrophages and dendritic cells that respond to the antigenic microbial components. TLR showed to play a crucial role in invoking innate immunity against the pathogenic invasion by inducing the production of cytokines upon binding to bacterial lipopolysaccharide in bovine mammary and endometrium epithelial cells [79, 80]. The Toll-like receptor signaling pathway was significantly over-represented in Red Sindhi, Tharparkar, Achai, Bhagnari, and Lohani by a total of thirty-nine—high or moderate impact variants harboring-genes, suggesting that most of the Pakistani-indicine breeds are evolving rapidly to adopt high or moderate impact changes in their immunity associate genes to cope with the infectious-environmental challenges in the tropical region.

The GO over-representation results should be very carefully evaluated because of the small number of samples used for each breed in this study. However, these results provide important genomic information regarding the economically important over-represented biological processes in all sequenced breeds.

## Conclusion

This study presented extensive genome analysis of eleven indigenous Pakistani cattle breeds following whole-genome resequencing using BGISEQ-500 sequencing platform. The selected low to medium coverage resequencing method lead to detection of 67,303,469 SNPs and 1,083,842 InDels in all studied cattle samples. The novel SNPs deposition to the dbSNP has considerably increased the number of indicine variants, which could play an important role towards the development of biased free SNP-array for genomic selection and genome-wide-association studies in indicine cattle. Coding and regulatory SNPs based genome comparison of five major and geographically diverse indicine breeds from Pakistan indicated that, the second best milking breed Red Sindhi is closely related to the best milking breed Sahiwal by sharing highest number of SNPs (173,344). On the other hand, the indicine breeds adapted to dry and extremely hot climate, Bhagnari and Tharparkar (white to gray coat color) shared highest number of SNPs (254,633). For all samples, variant function annotation revealed only 3,194 and 745 high impact (disruptive) SNPs and InDels, respectively. A total of 531 disruptive genes (256 genes harboring LoF SNPs and 275 harboring frame-shift) were used separately for GO enrichment analysis. The GO enrichment analysis revealed that most of the altered genes are significantly enriched in economically important biological processes, such as immune responses, heat tolerance, signaling pathways, cellular development and sensory perceptions. Therefore, this study provides baseline data for further research to reveal molecular mechanisms and identify potential genomic markers associated with economically important cattle traits for genomic selection (breeding), particularly in indicine breeds.

## Supporting information

S1 Table Summary of the total number of identified SNPs and change rate for all samples. (DOCX)

S2 Table A full list of analysis accession numbers for all 20 VCFs. (DOCX)

S3 Table High impact variants in all *Bos indicus* cattle from Pakistan. (DOCX)

S1 File Bull sources and introduction of Pakistani-indicine breed. (DOCX)

S2 File Detailed available descriptions of all over represented KEGG pathways found significantly enriched in Pakistani indicine breeds. (DOCX)

S1 Data All identified genes enriching KEGG pathways in Pakistani indicine breeds. (XLSX)
