# Supplementary material for: Genomic variants identified from whole-genome resequencing of indicine cattle breeds from Pakistan
Source: PLoS One. 2019 Apr 11;14(4):e0215065. doi: 10.1371/journal.pone.0215065 (PMC6459497; doi:10.1371/journal.pone.0215065)
Supplement: S1 Table — (DOCX) [file pone.0215065.s001.docx]

**S1 Table. Summary of the total number of identified SNPs and change rate for all samples**

| Breed | Animals ID | Genome effective  length | Total SNP | Change rate |
| --- | --- | --- | --- | --- |
| Achai | 64MCA | 2660906405 | 2800530 | 950 |
| Achai | 7CA | 2660906405 | 3121453 | 852 |
| Bhagnari | BN_18 | 2660906405 | 3244057 | 820 |
| Bhagnari | BN_20 | 2660906405 | 3835464 | 693 |
| Bhagnari | BN_23 | 2660906405 | 4070948 | 653 |
| Cholistani | 3702-C | 2660906405 | 2872502 | 926 |
| Cholistani | 6314-C | 2660906405 | 2394453 | 1111 |
| Dajal | Dajal C | 2660906405 | 3149645 | 844 |
| Dhanni | DH287 | 2660906405 | 3050129 | 872 |
| Dhanni | DH-363 | 2660906405 | 3691327 | 720 |
| Gabrali | G-27 | 2660906405 | 3020721 | 880 |
| Gabrali | G-3 | 2660906405 | 2829158 | 940 |
| Hisar Haryana | HH-44 | 2660906405 | 2826842 | 941 |
| Hisar Haryana | HH-46 | 2660906405 | 2523839 | 1054 |
| Lohani | Lohani-18 | 2660906405 | 4993244 | 532 |
| Red Sindhi | RS_303 | 2660906405 | 4519379 | 588 |
| Sahiwal | Zaibee | 2660906405 | 2572894 | 1034 |
| Sahiwal | Sunny | 2660906405 | 5205100 | 511 |
| Tharparkar | TH-138 | 2660906405 | 3155764 | 843 |
| Tharparkar | TH-158 | 2660906405 | 3426020 | 776 |
| Average change rate | | | | 827 |
