# Supplementary material for: Genomic variants identified from whole-genome resequencing of indicine cattle breeds from Pakistan
Source: PLoS One. 2019 Apr 11;14(4):e0215065. doi: 10.1371/journal.pone.0215065 (PMC6459497; doi:10.1371/journal.pone.0215065)
Supplement: S2 Table — (DOCX) [file pone.0215065.s002.docx]

**S2 Table. A full list of analysis accession numbers for all 20 VCFs.**

| Alias | Analysis accession |
| --- | --- |
| Gabrali-(Tag-G-3)_GW_Var_analysis | ERZ776599 |
| Dajal-(Tag-Dajal_C)_GW_Var_analysis | ERZ776600 |
| Bag_Narhi_Master-(Tag-BNM-male)_GW_Var_analysis | ERZ776601 |
| Achai-(Tag-64MCA)_GW_Var_analysis | ERZ776602 |
| Cholistani-(Tag-6314)_GW_Var_analysis | ERZ776603 |
| Lohani-(Tag-Lohani-18)_GW_Var_analysis | ERZ776604 |
| Cholistani-(Tag-3702-c)_GW_Var_analysis | ERZ776605 |
| Tharparkar-(Tag-TH-158)_GW_Var_analysis | ERZ776606 |
| Hisar-Hiryana-(Tag-HH-46)_GW_Var_analysis | ERZ776607 |
| Bhagnari-(Tag-BN_23)_GW_Var_analysis | ERZ776608 |
| Achai-(Tag-7CA)_GW_Var_analysis | ERZ776609 |
| Dhanni-(Tag-DH-363)_GW_Var_analysis | ERZ776610 |
| Hisar-Hiryana-(Tag-HH-44)_GW_Var_analysis | ERZ776611 |
| Sahiwal-(Tag-Sunny-2)_GW_Var_analysis | ERZ776612 |
| Sahiwal-(Tag-suny-1)_GW_Var_analysis | ERZ776613 |
| Tharparkar-(Tag-TH-138)_GW_Var_analysis | ERZ776614 |
| Dhanni-(Tag-DH287)_GW_Var_analysis | ERZ776615 |
| Bhagnari-(Tag-BN_18)_GW_Var_analysis | ERZ776616 |
| Red-Sindhi-(Tag-RedSindi_RS303)_GW_Var_analysis | ERZ776617 |
| Gabrali-(Tag-G-27)_GW_Var_analysis | ERZ776618 |
| Bhagnari-(Tag-BN_20)_GW_Var_analysis | ERZ776619 |
