# Supplementary material for: Genomic variants identified from whole-genome resequencing of indicine cattle breeds from Pakistan
Source: PLoS One. 2019 Apr 11;14(4):e0215065. doi: 10.1371/journal.pone.0215065 (PMC6459497; doi:10.1371/journal.pone.0215065)
Supplement: S3 Table — (DOCX) [file pone.0215065.s003.docx]

**S3 Table. High impact variants in all *Bos indicus* cattle from Pakistan.**

| High impact | Count | Affecting total genes | Affecting known genes |
| --- | --- | --- | --- |
| SNPs | 3194 | 462 | 265 |
| InDels | 745 | 275 | 168 |
