# Supplementary material for: Genomic variants identified from whole-genome resequencing of indicine cattle breeds from Pakistan
Source: PLoS One. 2019 Apr 11;14(4):e0215065. doi: 10.1371/journal.pone.0215065 (PMC6459497; doi:10.1371/journal.pone.0215065)
Supplement: S1 File — (DOCX) [file pone.0215065.s004.docx]

# Supplementary info file 1

# Bull sources and introduction of Pakistani-indicine breed

## Sources of Bulls

| **Breed** | **Location** |
| --- | --- |
| Sahiwal | Jahangirabad Cattle Farm (Wattoo farm) Khanewal |
| Cholistani | Livestock Station, Jugait Peer, Bahawalpur |
| Dhanni | Barani Livestock Production Research Institute, Khairi Murat, Attock |
| Tharparker | Livestock Experimental Station, Rakh Mahni, Tehsil Mankera, District Bhakkar |
| Achai | Livestock Farm Lower Dir |
| Gabrali | Gabral, near Kalam, district Swat |

## Introduction of indicine breeds of Pakistan

All indigenous cattle breeds of Pakistan are humped; therefore belong to the species *Bos indicus* (Khan *et al*., 2008). These breeds have natural resistance to various infectious and parasitic diseases. Among them, Sahiwal is the most prominent breed and is considered as the “best zebu milch breed in the tropics”. It is famous for its heat tolerance and adequate performance under subsistence production system. The breed is named after Sahiwal district, which is part of its hometract (Rehman *et al*., 2014, Ilatsia *et al*., 2012). Due to heavy dewlap, it is also known as 'Lola' (loose skin).

After Sahiwal, Red Sindhi is the reputed heat tolerant milch cattle breed originated from Sindh province of Pakistan (Panetto *et al*., 2017). The breed is of distinct red colour and darker than Sahiwal. Red shades vary from dark red to dim yellow but most commonly, the animals are dark red. Horns are thick at the base and emerge laterally and curve upward.

Bhagnari breed is the off shoot of Brahman cattle (*Bos indicus*), having the fore fathers of Gir, Kankrej, Gujarat, and Ongole with 25% blood contribution of each breed (Ali *et al*., 2018). Besides livelihood, farmers keep Bhagnari breed as symbol of their pride (Akhtar *et al*., 2015). Bhagnari cattle are considered as potential beef producer, due to which this breed was crossed with Australian Drought Master (*Bos taurus*) to develop first ever beef breed of Pakistan i.e. “Nari Master” (Kakar *et al*., 2017).

Dajal breed originates from Dajal area in Dera Ghazi Khan. Dajal breed is considered as an offshoot of the Bhagnari breed, having similar physical features but it is comparatively smaller in size and lighter in colour (Tahir, 1997).

Dhanni breed, also known as Pothwari due to its hometract which is Attock, Rawalpindi and Jhelum districts. The coloring in the majority of Dhanni cattle consists of black or red/brown spots on a white coat. It is a sturdy animal having great agility. Therefore, it is frequently seen racing in locally popular bull cart races (Tahir, 1997).

Lohani breed is found in the Loralai district of Baluchistan Province. They have a red coat splashed with white spots. They have a short-stature, well developed hump, short thick horns, short neck, small ears, moderate dewlap and black switch of tail. It is a hardy and sure-footed animal due to which bulls are suitable for light work in hilly and sub-hilly areas (Tahir, 1997).

Tharparkar is a dual purpose cattle breed. The breed is also known as Thari, after the desert of Thar. The breed is medium sized compact with white and light grey coloured coat. Face and extremities are darker than rest of the body. In bulls neck, hump, and fore and hind quarters are also dark. Tharparkar breed came into prominence during the first World War when some animals were taken to supply milk for the Near East army camps. Here their capacity for production under rigorous feeding and unfavorable environmental conditions at once became apparent (Joshi and Phillips 1953; Mason, 1996).

Cholistani is also a dual purpose breed which is found in Cholistan desert in Bahawalpur, Pakistan. Cholistani animals are usually speckled red, brown or black. These animals have been derived from the crossing of [Sahiwal](http://afs.okstate.edu/breeds/cattle/sahiwal/index.html) with the local cattle (Mason, 1996).

Achai and Gabrali are small-sized cattle breeds found in Khyber Paktunkhwa, particularly adjoining parts of Afghanistan. These animals are suitable for mountainous terrain and can resist cold as well as warm climate. Due to their small body, they have the ability to thrive under scarce fodder availability and can produce adequately under a hilly and sub-hilly subsistence production setup (Uddin *et al*., 2014).

For further details please visit <http://afs.okstate.edu/breeds/cattle>.

## References:

1. Rehman Z, Khan MS, Mirza MA. Factors affecting performance of Sahiwal cattle–a review. The Journal of Animal & Plant Sciences. 2014 Jan 1;24(1):1-2.
2. Ilatsia ED, Roessler R, Kahi AK, Piepho HP, Zárate V. Production objectives and breeding goals of Sahiwal cattle keepers in Kenya and implications for a breeding programme. Tropical animal health and production. 2012 Mar 1;44(3):519-30.
3. Khan MS, Rehman Z, Khan MA, Ahmad S. Genetic resources and diversity in Pakistani cattle. Pak Vet J. 2008 Jan 1;28(2):95-102.
4. Panetto JD, Silva MV, Leite RM, Machado MA, Bruneli FA, Reis DD, Peixoto MG, Verneque RD. Red Sindhi cattle in Brazil: population structure and distribution. Embrapa Gado de Leite-Artigo em periódico indexado (ALICE). 2017 Mar 15.
5. Ali I, Tariq MM, Waheed A, Yousafzai FA, Bokhari FA, Rafeeq M, Ali M, Attique MA, Amin S, Hameed T. Exclusive Characteristics of the Bhag Nari Cattle among the Other Indigenous Cattle Breeds of Pakistan. Pakistan Journal of Zoology. 2018 Oct 31;50(5):1979-.
6. Akhtar, P., Kalsoom, U., Ali, S., Yaqoob, M., Javed, K., Babar, M.E., Mustafa, M.I. and Sultan, J.I., 2015. J. Anim. Plt. Sci., 22: 347-352
7. Kakar ME, Khan MA, Khan MS, Ashraf K, Kakar MA, Jan S, Razzaq A. Prevalence of tick infestation in different breeds of cattle in Balochistan. JAPS: Journal of Animal & Plant Sciences. 2017 Jun 1;27(3).
8. Muhammad Tahir, University of Agriculture, Faisalabad. 1997. Accessible at: <http://afs.okstate.edu/breeds/cattle/dhanni/index.html>
9. Joshi, N.R., Phillips, R.W. (1953) Zebu Cattle of India and Pakistan, FAO Agriculture Studies No. 19, Publ. by FAO, Rome, 256 pp.
10. Mason, I.L. 1996. A World Dictionary of Livestock Breeds, Types and Varieties. Fourth Edition. C.A.B International. 273 pp.
11. Uddin H, Khan HU, Khan MI, Khan R, Naveed A. (2014). Productive and reproductive performance of achai cattle maintained at Livestock Research & Development Station Surezai Peshawar, Pakistan. Journal of Animal Health and Production (Pakistan).
