# Supplementary material for: Genomic variants identified from whole-genome resequencing of indicine cattle breeds from Pakistan
Source: PLoS One. 2019 Apr 11;14(4):e0215065. doi: 10.1371/journal.pone.0215065 (PMC6459497; doi:10.1371/journal.pone.0215065)
Supplement: S2 File — (DOCX) [file pone.0215065.s005.docx]

# Supplementary information file 2

## KEGG biological pathways found enriched by genes harboring high or moderate impact SNVs in Pakistani indicine breeds

Following are the detailed available descriptions of all over represented KEGG pathways in Pakistani indicine breeds, retrieved from <https://www.genome.jp/kegg/pathway.html>;

### Wnt signaling pathway - Bos taurus (cow) bta04310:

Description

Wnt proteins are specialized secretory proteins that play role in different cellular developmental processes by promoting morphogenesis, differentiation of progenitor-cells into more specific types, cell fate specification, and controlling irregular cell divisions in different tissues and organs. There are at least three different Wnt pathways: the canonical pathway, the planar cell polarity (PCP) pathway and the Wnt/Ca2+ pathway. In the canonical Wnt pathway, the major effect of Wnt ligand binding to its receptor is the stabilization of cytoplasmic beta-catenin through inhibition of the bea-catenin degradation complex. Beta-catenin is then free to enter the nucleus and activate Wnt-regulated genes through its interaction with TCF (T-cell factor) family transcription factors and concomitant recruitment of coactivators. Planar cell polarity (PCP) signaling leads to the activation of the small GTPases RHOA (RAS homologue gene-family member A) and RAC1, which activate the stress kinase JNK (Jun N-terminal kinase) and ROCK (RHO-associated coiled-coil-containing protein kinase 1) and leads to remodelling of the cytoskeleton and changes in cell adhesion and motility. WNT-Ca2+ signalling is mediated through G proteins and phospholipases and leads to transient increases in cytoplasmic free calcium that subsequently activate the kinase PKC (protein kinase C) and CAMKII (calcium calmodulin mediated kinase II) and the phosphatase calcineurin.

### Vascular smooth muscle contraction - Bos taurus (cow) bta04270:

Description

The vascular smooth muscle cell (VSMC) is a specialized contracting cell required for the regulation of blood flow and pressure in different stress conditions, by adjusting the diameter of the blood vessels. The principal mechanisms that regulate the contractile state of VSMCs are changes in cytosolic Ca2+ concentration ([Ca2+]c). In response to vasoconstrictor stimuli, Ca2+ is mobilized from intracellular stores and/or the extracellular space to increase [Ca2+]c in VSMCs. The increase in [Ca2+]c, in turn, activates the Ca2+-CaM-MLCK pathway and stimulates MLC20 phosphorylation, leading to myosin-actin interactions and, hence, the development of contractile force. The sensitivity of contractile myofilaments or MLC20 phosphorylation to Ca2+ can be secondarily modulated by other signaling pathways. During receptor stimulation, the contractile force is greatly enhanced by the inhibition of myosin phosphatase. Rho/Rho kinase, PKC, and arachidonic acid have been proposed to play a pivotal role in this enhancement.

The signaling events that mediate relaxation include the removal of a contractile agonist (passive relaxation) and activation of cyclic nucleotide-dependent signaling pathways in the continued presence of a contractile agonist (active relaxation). Active relaxation occurs through the inhibition of both Ca2+ mobilization and myofilament Ca2+ sensitivity in VSMCs.

### VEGF signaling pathway - Bos taurus (cow) bta04370:

Description

Vascular endothelial growth factor (VEGF) signaling pathway is a VEGF to VEGFR-2 binding dependent cascade of chemical reactions that play a crucial role in both pathologic and physiologic formation of blood vessels, called angiogenesis. VEGFR-2 is a major mediator of VEGF driven response in endothelial cells. The VEGFR-2 to VEGF binding initiates various signaling transduction pathways leading to endothelial cell proliferation and subsequent migration to ensure their survival and vascular permeability. For example, the binding of VEGF to VEGFR-2 leads to dimerization of the receptor, followed by intracellular activation of the PLCgamma;PKC-Raf kinase-MEK-mitogen-activated protein kinase (MAPK) pathway and subsequent initiation of DNA synthesis and cell growth, whereas activation of the phosphatidylinositol 3' -kinase (PI3K)-Akt pathway leads to increased endothelial-cell survival. Activation of PI3K, FAK, and p38 MAPK is implicated in cell migration signaling.

### HIF-1 signaling pathway - Bos taurus (cow) bta04066:

Description

Hypoxia-inducible factor 1 (HIF-1) is a transcription factor that functions as a master gene regulator of numerous hypoxia-inducible protein-coding genes under hypoxic condition. The HIF-1 targeted genes encode proteins that facilitate Oxygen supply in response to Oxygen depletion. It consists of two subunits: an inducibly-expressed HIF-1alpha subunit and a constitutively-expressed HIF-1beta subunit. Under normoxia, HIF-1 alpha undergoes hydroxylation at specific prolyl residues which leads to an immediate ubiquitination and subsequent proteasomal degradation of the subunit. In contrast, under hypoxia, HIF-1 alpha subunit becomes stable and interacts with coactivators such as p300/CBP to modulate its transcriptional activity. Despite its name, HIF-1 is induced not only in response to reduced oxygen availability but also by other stimulants, such as nitric oxide, or various growth factors.

### Toll-like receptor signaling pathway - Bos taurus (cow) bta04620:

Description

Toll-like receptors (TLRs) are specific families of pattern recognition receptors, responsible for detecting microbial pathogens and produce innate immune responses to infectious microbes. Mammalian TLRs are membrane-bound receptors, expressed on macrophages and dendritic cells that respond to the antigenic microbial components. Pathogen recognition by TLRs provokes rapid activation of innate immunity by inducing production of pro-inflammatory cytokines and up-regulation of co-stimulatory molecules. TLR signaling pathways are separated into two groups: a MyD88-dependent pathway that leads to the production of pro-inflammatory cytokines with quick activation of NF-{kappa}B and MAPK, and a MyD88-independent pathway associated with the induction of IFN-beta and IFN-inducible genes, and maturation of dendritic cells with slow activation of NF-{kappa}B and MAPK.

### ECM-receptor interaction - Bos taurus (cow) bta04512:

Description

The extracellular matrix (ECM) is a mixture of complex structural and functional macromolecules that maintain the structure and function of cells and tissue, with an essential role in the morphogenesis of tissue and organ. The ECM interaction with the transmembrane cellular molecules can directly or indirectly control the cellular migration, adhesion, proliferation, apoptosis and differentiation. Specific interactions between cells and the ECM are mediated by transmembrane molecules, mainly integrins and perhaps also proteoglycans, CD36, or other cell-surface-associated components. In addition, integrins function as mechanoreceptors and provide a force-transmitting physical link between the ECM and the cytoskeleton. Integrins are a family of glycosylated, heterodimeric transmembrane adhesion receptors that consist of noncovalently bound alpha- and beta-subunits.

### JAK-STAT signaling pathway - Bos taurus (cow) bta04630:

Description

In mammals, the Janus kinase/signal transducers and activators of transcription (JAK/STAT) pathway is the major signaling mechanism for a wide array of cytokines and growth factors that transduces a multitude of signals and modulate the expression of target genes for the development and homeostasis in animals, from humans to flies. Following the binding of cytokines to their cognate receptor, STATs are activated by members of the JAK family of tyrosine kinases. Once activated, they dimerize and translocate to the nucleus and control the target genes expression. In addition to the activation of STATs, JAKs mediate the recruitment of other molecules such as the MAP kinases, PI3 kinase etc. These molecules process downstream signals via the Ras-Raf-MAP kinase and PI3 kinase pathways which results in the activation of additional transcription factors.
